# Application of the Boruta algorithm to assess the multidimensional determinants of malnutrition among children under five years living in southern Punjab, Pakistan

**DOI:** 10.1186/s12889-024-17701-z

**Published:** 2024-01-12

**Authors:** Javeria Saleem, Rubeena Zakar, Muhammad Salman Butt, Rana Muhammad Aadil, Zulfiqar Ali, Gul Mehar Javaid Bukhari, Muhammad Ishaq, Florian Fischer

**Affiliations:** 1 Department of Public Health, University of the Punjab, Lahore, Pakistan; 2 National Institute of Food Sciences and Technology, University of Agriculture, Faisalabad, Pakistan; 3 College of Statistical Sciences, University of the Punjab, Lahore, Pakistan; 4 Department of Community Medicine, Rawalpindi Medical University, Rawalpindi, Pakistan; 5 Department of Sociology, Institute of Social and Cultural Studies, University of the Punjab, Lahore, Pakistan; 6 Institute of Public Health, Charité– Universitätsmedizin Berlin, Berlin, Germany

**Keywords:** Malnutrition, Undernutrition, Stunting, Wasting

## Abstract

**Background:**

Malnutrition causes nutrient deficiencies that have both physical and clinical consequences in severe acute malnutrition children. Globally, there were 47 million wasted children under the age of five in 2019. One in four were located in sub-Saharan Africa, with half being in South Asia. This study aims to apply the Boruta algorithm to identify the determinants of undernutrition among children under five living in Dera Ghazi Khan, one of the marginalized districts of densely populated Punjab Province in Pakistan.

**Methods:**

A multicenter cross-sectional study design was used to collect data from 185 children with severe acute malnutrition aged under five years visiting the OTPs centers located in Dera Ghazi Khan, Punjab, Pakistan. A purposive sampling technique was used to collect data using a pretested structured questionnaire from parents/caregivers regarding family sociodemographic characteristics, child nutrition, and biological and healthcare characteristics. Anthropometric measurements, including height, weight, and mid-upper arm circumference, were collected. The Boruta models were used to incorporate the children’s anthropometric, nutritional, and household factors to determine the important predictive variables for undernutrition using the Boruta package in R studio.

**Results:**

This study included 185 children, with a mean age of 15.36 ± 10.23 months and an MUAC of 10.19 ± 0.96 cm. The Boruta analysis identifies age, mid-upper arm circumference, weaning practices, and immunization status as important predictors of undernutrition. Income per month, exclusive breastfeeding, and immunization status were found to be key factors of undernutrition in children under the age of five.

**Conclusion:**

This study highlights age, mid-upper arm circumference, weaning practices, and immunization status as key determinants of weight-for-height and weight-for-age in children under five years. It also suggests that economic context may influence undernutrition. The findings can guide targeted strategies for combating undernutrition.

## Introduction

Malnutrition causes nutrient deficiencies that have both physical and clinical consequences in severe acute malnutrition children. Stunting is a form of undernutrition that is characterized by a child’s height for age Z score (SD ≤ 2) [1]. Wasting is characterized by low weight for height and poses a greater risk than stunting [2]. Wasting and stunting are often described as “acute” and “chronic” undernutrition, respectively. Underweight is characterized as a low weight-for-age. Underweight children might be stunted, wasted, or both [3]. Globally, there were 47 million wasted children under the age of five in 2019. One in four were located in sub-Saharan Africa, with half being in South Asia. South Asia had almost two out of every five stunted children [4].

Malnutrition still affects young children under the age of five in developing countries such as Pakistan. In Pakistan, 12 million children are stunted, with a national average of 40.2%. Stunting rates in Pakistan remain globally critical, with a slow reduction rate. Wasting among young children is increasing and had a prevalence of 17.7% in 2017, leading to a nutrition emergency in Pakistan [5]. Malnutrition is a major cause of intergenerational starvation, destroying the future productivity of nations and raising the economic burden [6]. Studies conducted in low-income countries have shown that male children are more likely to be stunted than their female counterparts, with a high prevalence in sub-Saharan Africa. Some studies conducted in Asia have documented that female children are more vulnerable [7, 8].

Malnutrition is caused by several interrelated variables and has both acute and chronic negative health effects [9]. The key factors contributing to child undernutrition are political unpredictability, poverty, and a lack of education that leads to inadequate dietary intake. The most typical causes of undernutrition in children are conditions such as diarrhea and inflammatory bowel syndrome, which have an impact on children’s development and growth [10, 11]. Child malnutrition can be influenced by several variables, such as fetal growth retardation, a lack of exclusive breastfeeding, incorrect complementary feeding, recurrent illnesses, food scarcity, and vitamin deficiencies [10, 12].

Malnutrition is the main cause of morbidity and mortality in children under five, and Pakistan ranks 22nd in the world for underfive child mortality [13]. Pakistan has recently faced natural disasters such as floods, famine/drought, and earthquakes [14]. A study of 656 households in the flood-affected areas of Pakistan found that 40.5% of children suffer from stunting, with factors such as age, maternal age, family type, water quality, and toilet facilities [15]. Similarly, a study in Khyber Pakhtunkhwa, Pakistan, revealed that 46% of children suffer from MUAC-based malnutrition, highlighting the need for targeted community-based nutrition awareness programs [16]. The most concerning effect of these catastrophes is the rise in childhood malnutrition. Previous studies have focused on explaining poverty and low socioeconomic status as the main cause of malnutrition in Pakistan.

The present study used the Boruta algorithm, a novel approach to studying malnutrition determinants, to fill a gap in the literature by focusing on southern Punjab, Pakistan. The algorithm’s ability to handle high-dimensional data and its ability to identify key determinants could guide more effective policies. This study is critical for comprehending the current situation with undernutrition among children under 5 years. The integration of diverse data sources, such as socioeconomic, health, and environmental data, could provide a more holistic understanding of malnutrition and undernutrition in particular. The possible contributions of the study to policy might highlight its importance.

Malnutrition can be caused by a variety of variables and requires creating an effective response strategy. The policy environment significantly influences the implementation and efficacy of nutrition interventions. Understanding the malnutrition framework is crucial for policy formulation and strategic actions, but its translation depends on a strong local understanding of subcomponents and their interactions. Previous research has typically used only univariate methods to identify the determinants of malnutrition [17, 18]. Recent research has employed the Boruta relevant feature selection wrapper algorithm to identify the key predictive determinants for undernutrition [19]. The Boruta algorithm compares the original attributes’ importance with random, permuted copies and eliminates irrelevant features [20].

Undernutrition in children under five requires a comprehensive public health approach, addressing factors such as illiteracy, poverty, and poor living standards through policies and awareness. Undernutrition treatments still cause significant morbidity and mortality, necessitating effective interventions to prevent millions of child deaths annually and contribute to sustainable development goals. This study aims to apply the Boruta algorithm to identify the important predictors of undernutrition among children under five living in Dera Ghazi Khan, which is one of the marginalized districts of the densely populated Punjab province in Pakistan.

## Materials and methods

### Study design and setting

A multicenter cross-sectional study was carried out for data collection during March 2021 to June 2022. The “National Program for Family Planning and Primary Health Care” operated four functional outpatient therapeutic program centers (OTPs) at basic health units (BHUs), including Samina, Jhokutra, and Aaliwala, and one OTP at a rural health center (RHC), Kotchutta, of Dera Ghazi Khan, Punjab, Pakistan, for the collection of children with severe acute malnutrition. These four centers were selected because they were more active than the others, with an appropriate number of workers, patients, and therapeutic food. Participants in the study were selected from these government-designated centers for the treatment of malnutrition. These chosen areas of Dera Ghazi Khan District are underdeveloped, with poor socioeconomic situations, poverty, insecure housing tenure, overpopulation, and unclean living conditions.

### Study population

A purposive sampling technique was used to recruit children aged 6–59 months living in district Dera Ghazi Khan diagnosed with “severe acute malnutrition” without chronic comorbidities. A mid-upper arm circumference (MUAC) of ≤ 11.5 cm, a weight for height Z score of -3, or bilateral edema of grades 1–2 were used as the World Health Organization-suggested inclusion criteria for severe acute malnutrition [21]. All of the children had good general health, and they shared the same racial and language background. Parents/caregivers were informed of the study’s purpose and provided with a written consent form to participate in the study. Children with severe malnutrition-related problems such as severe hypoglycemia, severe anemia, severe pitting edema, anorexia, hypothermia, or high pyrexia were excluded.

### Sample size

**Fig. 1:**
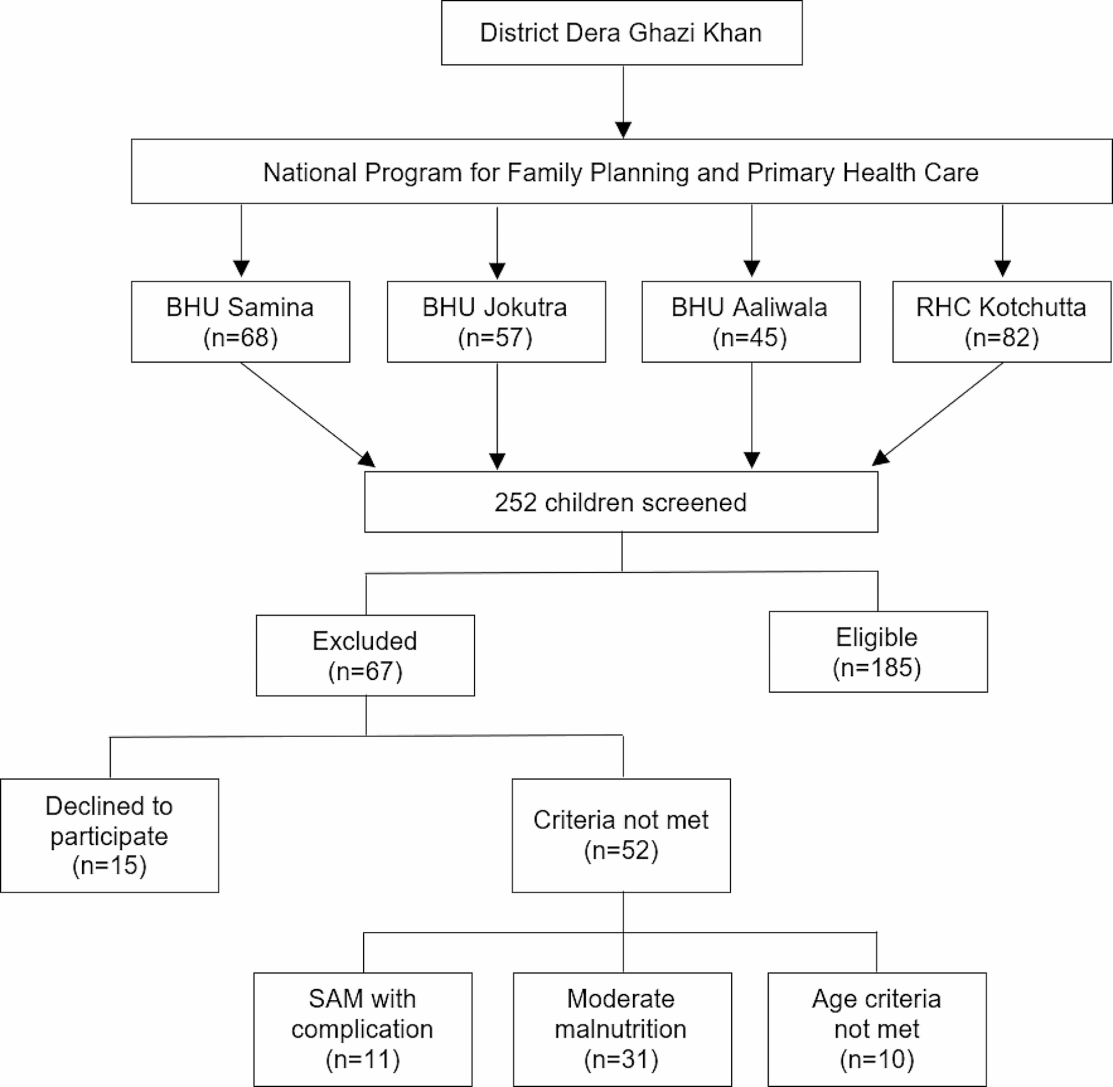
Flowchart of sample selection

The cross-sectional study formula was used to determine the sample size, with the error term (d) set at 0.05 and the prevalence of severe malnutrition (p) at 11%. A sample size of 151 was calculated using the following formula (Eq. 1), and this number was raised to 185 to increase study precision, strength, and accuracy. A total of 252 children were screened for participation in this study, with 67 being excluded due to not satisfying the inclusion criteria (Fig. 1).

1 $$ n={Z}^{2}. P(1-P)/{d}^{2}$$

### Data collection

Parents were interviewed at OTP centers using a structured questionnaire that had been pretested to gather information about family sociodemographics (income, parental education, occupation, household size, and family structure), infant and young child feeding (IYCF), anthropometric measurements (height, weight, and MUAC) and healthcare characteristics (child medical history, immunization record, and access to healthcare). Income from all sources in Pakistani rupees (PKR) was used to estimate the monthly income of the family.

In accordance with WHO recommendations, complementary feeding practices (amount, variety, and frequency) and exclusive breastfeeding were evaluated [22]. The vaccination record served as evidence that the Expanded Program of Immunization (EPI) schedule for child immunization had been followed [23]. The child’s medical history was assessed to identify wasting, stunting, and underweight. Parents/caregivers provided a history to identify seizure disorders and neurological deficits in children; sick children were excluded.

For children who had hospital deliveries, the gestational age was derived from the antenatal record; in cases of home deliveries, it was based on the mother’s narrative. The number of lost gestational weeks was deducted from the present age to account for premature birth in children up to 24 months of age (37 weeks gestation).

Nutritional supervisors conducted anthropometric measurements, recording double anthropometric values if they differed from one another. Children’s weight was assessed without clothing or in light outfits using SECA 336 baby scales, while children 2 years or older were weighted using SECA 874 Electronic Scale. Infant/child weights were calculated as the difference between these two measures and were close to 10 g.

A length-measuring board with a fixed headrest and an adjustable foot piece (SECA GmbH & Co. KG, Hamburg, Germany) was used to measure the recumbent length of children under 87 cm in height to the nearest 0.1 cm. Children who were taller than 87 cm were measured by having them stand with their heels touching a flat horizontal plate attached to the measurement board. The WHO Child Growth Standards were used to generate the weight-for-height Z scores and the height-for-age Z scores [1]. WHO ANTHRO, version 3.2.2, was used to classify the nutritional status of the children.

### Data analysis

The demographic and household characteristics are presented as categorical variables to show the frequency distribution by using IBM SPSS 25.0. In addition, for statistical analyses, we used the Boruta package, which is available from the comprehensive R archive network at https://cran.r-project.org/web/packages/Boruta/index.html. The determinants were identified using the Boruta feature selection, and the variables’ importance scores were calculated for weight-for-height, weight-for-age, and undernutrition. Undernutrition data generated from the Boruta outcome are presented in the graphs. Blue boxplots represent a shadow attribute’s minimum, average, and maximum Z scores for the undernutrition determinants. Z scores of qualities that were rejected are presented in red and confirmed are presented in green boxplots. The attStats function also generates a data frame with Z score statistics for each attribute.

### Ethics approval

The provincial and district health departments of Punjab, Pakistan, gave their approval for the study to be conducted. The University of Punjab in Pakistan’s Ethical Review and Advanced Study Research Board (ref-9/2352-ACAD) gave its approval to all procedures involving human subjects during this study, which was carried out following the Declaration of Helsinki’s principles [24]. All subjects provided written consent to participate in the study.

## Results

This study included 185 children, with a mean age of 15.36 ± 10.23 months, who had a MUAC of 10.19 ± 0.96 cm. In terms of gender, the study included 81 male participants (43.8%) and 104 female participants (56.2%). When considering monthly income, the majority of participants (69.2%) had an income of ≤ 15,000 PKR, while 30.8% had an income ranging from 15,000 to 35,000 PKR. Among the study participants, 22.7% reported a history of a parasite, whereas the majority (77.3%) had no such history. In terms of TB, 43.8% of the participants had a history of TB. Additionally, 15.1% of the participants reported a history of measles. The participants experienced varying numbers of illness episodes, with 54.1% reporting 1–7 episodes and 45.9% having 8–15 episodes. Only 19.5% of the mothers exclusively breastfed their infants, whereas the majority (80.5%) did not. In addition, 89.2% of the subjects had inadequate complementary feeding practices, as shown in Table 1.

**Table 1:** Characteristics of the study participants

Determinant code	Variable	Category	n (%)
D1	Age (15.36 ± 10.23 Months)	≤ 12 months	112 (60.5)
		13–24 Months	45 (24.3)
		≥ 25 Months	28 (15.1)
D2	Gender	Male	81 (43.8)
		Female	104 (56.2)
D3	Mid Upper Arm Circumference (MUAC) (10.19 ± 0.95 cm)	≤ 10 cm	99 (53.5)
		≥ 10.1 cm	86 (46.5)
D4	Income per month	≤ 15,000 PKR	128 (69.2)
		15,000–35,000 PKR	57 (30.8)
D5	History of parasite	Yes	42 (22.7)
		No	143 (77.3)
D6	History of measles	Yes	28 (15.1)
		No	157 (84.9)
D7	History of scabies	Yes	28 (15.1)
		No	157 (84.9)
D8	History of TB	Yes	81 (43.8)
		No	104 (56.2)
D9	Exclusive breastfeeding	Yes	36 (19.5)
		No	149 (80.5)
D10	Weaning practices	Adequate	45 (24.3)
		Nonadequate	140 (75.7)
D11	Immunization	Incomplete	46 (24.9)
		Complete	139 (75.1)
D12	Father’s education	Primary & above	73 (39.5)
		No education	112 (60.5)
D13	Mother’s education	Primary & above	52 (28.1)
		No education	133 (71.9)
D14	Hygiene	Good	20 (10.8)
		Poor	165 (89.2)
D15	Underfive siblings	≤ 2	144 (77.8)
		≥ 3	41 (22.2)
D16	Household size	1–10	23 (12.4)
		≥ 10	162 (87.6)

**Table 2:** Determinants importance scores from Boruta algorithm for weight-for-height

Determinants	Meanimp	Medianimp	Minimp	Maximp	Normhits	Decision
Age	7.11	7.02	2.20	11.61	0.93	Confirmed
Gender	1.56	1.28	-0.71	4.08	0.03	Rejected
MUAC	15.69	15.88	9.99	21.18	1.00	Confirmed
Income per month	2.56	2.37	-1.31	6.62	0.43	Tentative
History of Parasites	-0.97	-1.08	-2.83	0.66	0.00	Rejected
History of Measles	-0.45	-0.48	-1.93	1.27	0.00	Rejected
History of Scabies	-0.11	0.68	-3.82	1.46	0.00	Rejected
History of TB	0.00	-0.22	-0.80	1.15	0.00	Rejected
Exclusive feeding	-0.63	-0.48	-3.22	0.78	0.00	Rejected
Weaning practices	4.70	4.74	0.20	9.41	0.73	Confirmed
Immunization	4.16	4.08	-0.09	8.22	0.70	Confirmed
Father Education	0.11	-0.02	-2.12	1.82	0.00	Rejected
Mother Education	0.72	0.65	-2.17	4.04	0.02	Rejected
Hygiene	1.93	1.95	-1.05	4.52	0.07	Rejected
Under5-siblings	1.22	1.17	-1.16	3.06	0.00	Rejected
Household size	0.00	0.05	-1.61	1.52	0.00	Rejected

The Boruta analysis was conducted to identify the determinants that have significant importance in predicting weight-for-height among children under five years. The analysis revealed several key findings and suggested that age (D1) emerged as the most important determinant, consistently demonstrating high importance scores across multiple iterations. Mid-upper arm circumference (D3) also exhibited significant importance and was consistently identified as important in all iterations. Additionally, weaning practices (D10) and the immunization status of the children (D11) were found to be important determinants, as shown in Fig. 1a; Table 2.

**Fig. 2:**
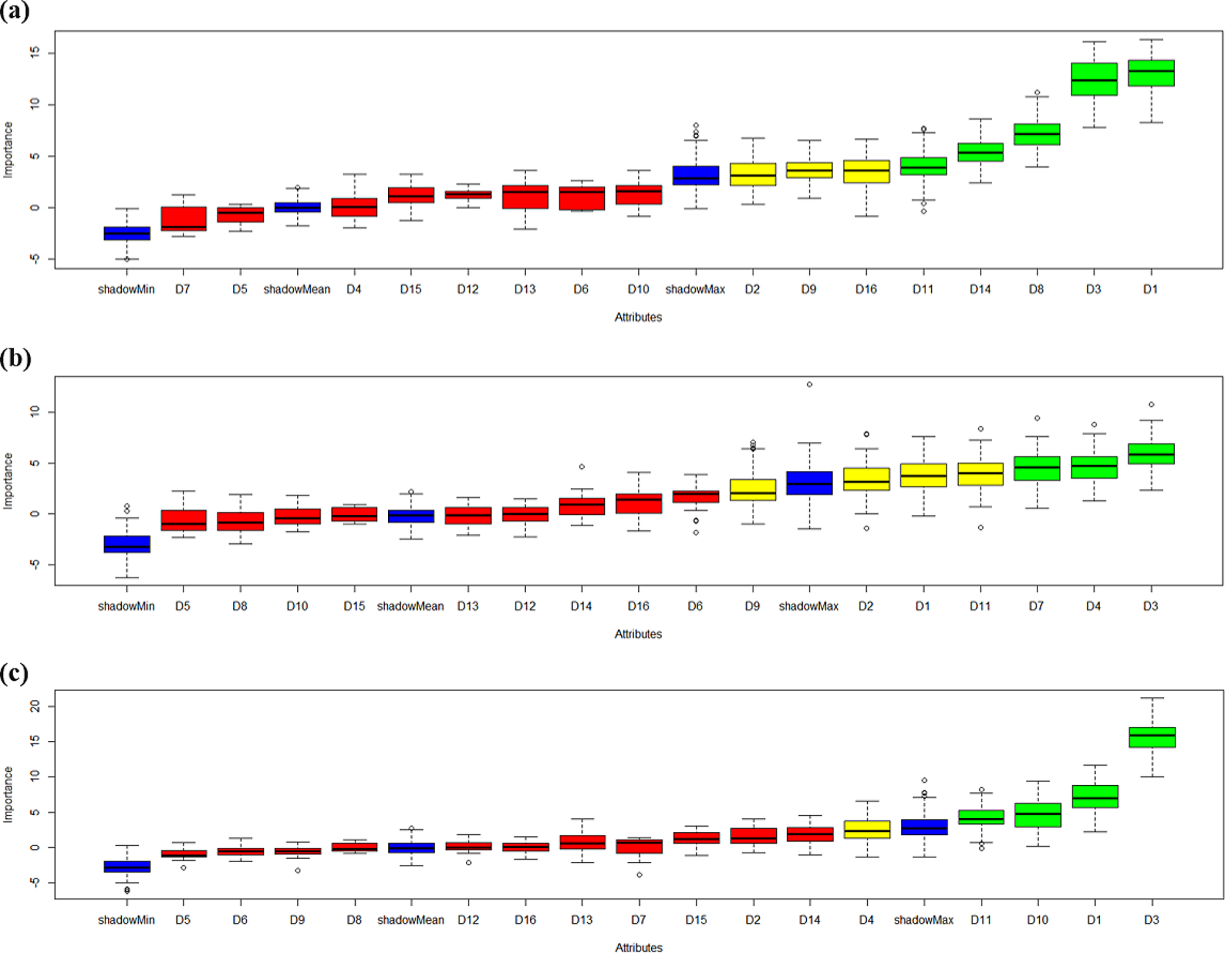
**a-c**: Variable importance scores from the Boruta algorithm for (**a**) weight-for-height, (**b**) weight-for-age, and (**c**) underweight. Blue boxplots represent a shadow attribute’s minimum, average, and maximum Z scores. Z scores of qualities that were rejected and confirmed, respectively, are represented by red and green boxplots

However, income per month (D4) displayed a moderate importance score and was labelled tentative, requiring further investigation for confirmation. On the other hand, determinants such as gender (D2), history of a parasite (D5), history of measles (D6), history of scabies (D7), history of TB (D8), exclusive breastfeeding (D9), father’s education (D12), mother’s education (D13), hygiene (D14), under five siblings (D15), and household size (D16) were rejected as unimportant, as shown in Fig. 2a; Table 2. These findings provide valuable insights into the factors influencing weight-for-height and can inform future research and modelling efforts in this domain.

The study also aimed to determine the important determinants for predicting weight-for-age using the Boruta algorithm. Determinants MUUAC and income per month emerged as confirmed determinants, consistently demonstrating significant importance in multiple iterations. A history of scabies also showed consistent importance and was confirmed as a significant determinant. On the other hand, age, sex, exclusive feeding and immunization displayed moderate importance and were labelled tentative, indicating the need for further investigation. The study found that factors such as history of parasites (D5), measles (D6), tuberculosis (D8), weaning practices (D10), parents’ education (D12, D13), hygiene (D14), under5-siblings (D15), and household size (D16) were not significant in predicting weight-for-age, as shown in Fig. 2b; Table 3. The Boruta analysis identified age, income per month, exclusive feeding, and immunization as significant determinants for predicting underweight, with gender and history of tuberculosis as tentative determinants. Rejected determinants had little influence, as shown in Fig. 2c; Table 4.

**Table 3:** Determinants importance scores from the Boruta algorithm for weight-for-age

Determinants	Meanimp	Medianimp	Minimp	Maximp	Normhits	Decision
Age	3.85	3.75	-0.21	7.64	0.61	Tentative
Gender	3.44	3.19	-1.40	7.87	0.58	Tentative
MUAC	5.97	5.88	2.33	10.78	0.92	Confirmed
Income per month	4.68	4.74	1.24	8.82	0.77	Confirmed
History of Parasites	-0.58	-1.02	-2.30	2.26	0.01	Rejected
History of Measles	1.58	1.97	-1.85	3.84	0.02	Rejected
History of Scabies	4.47	4.55	0.55	9.44	0.71	Confirmed
History of TB	-0.65	-0.85	-2.94	1.92	0.00	Rejected
Exclusive feeding	2.35	2.03	-0.97	7.01	0.41	Tentative
Weaning practices	-0.23	-0.40	-1.77	1.86	0.00	Rejected
Immunization	3.89	4.03	-1.35	8.38	0.63	Tentative
Father Education	-0.14	0.02	-2.26	1.45	0.00	Rejected
Mother Education	-0.27	-0.13	-2.12	1.61	0.00	Rejected
Hygiene	0.92	0.90	-1.13	4.66	0.02	Rejected
Under5-siblings	-0.04	-0.18	-1.02	0.95	0.00	Rejected
Household size	1.19	1.41	-1.70	4.07	0.03	Rejected

**Table 4:** Determinants importance scores from the Boruta algorithm for underweight

Determinants	Meanimp	Medianimp	Minimp	Maximp	Normhits	Decision
Age	4.60	4.57	1.56	8.90	0.72	Confirmed
Gender	3.23	3.01	-0.45	9.43	0.46	Tentative
MUAC	0.09	0.36	-1.44	2.33	0.00	Rejected
Income per month	5.02	4.97	-0.19	11.35	0.75	Confirmed
History of Parasites	-0.31	-0.08	-3.27	1.85	0.02	Rejected
History of Measles	0.78	0.67	-1.78	3.19	0.03	Rejected
History of Scabies	2.03	1.89	-2.33	5.52	0.14	Rejected
History of TB	3.45	3.54	0.20	7.13	0.54	Tentative
Exclusive feeding	5.63	5.70	0.91	12.00	0.83	Confirmed
Weaning practices	-0.38	-0.73	-2.44	3.26	0.01	Rejected
Immunization	4.82	5.14	-0.26	9.49	0.73	Confirmed
Father Education	3.85	3.66	0.26	8.24	0.65	Tentative
Mother Education	1.30	1.07	-1.03	4.18	0.04	Rejected
Hygiene	0.42	0.31	-1.27	2.23	0.00	Rejected
Under5-siblings	0.58	0.31	-1.32	2.40	0.02	Rejected
Household size	2.00	2.22	-1.22	5.00	0.19	Rejected

## Discussion

The present study aimed to identify the important determinants for predicting weight-for-height, weight-for-age, and underweight using the Boruta algorithm. The findings from the Boruta analysis provided valuable insights into the factors influencing these anthropometric measurements. The Boruta algorithm was chosen for this analysis due to its robustness in handling multiple features, capturing nonlinear pattern relationships, and providing more robust discriminant power than classical statistics. For weight-for-height, the Boruta analysis identified age, MUAC, weaning practices, and immunization as confirmed determinants. Age, with the highest importance score, consistently demonstrated its significance across multiple iterations. For weight-for-age, the Boruta analysis revealed MUAC, income per month, and history of scabies as confirmed determinants. MUAC and income per month consistently displayed significant importance scores, highlighting their influence on weight-for-age prediction. In the case of undernutrition, the Boruta analysis confirmed age, income per month, exclusive feeding, and immunization as important determinants. Age, income per month, and immunization consistently demonstrated significant importance, suggesting their strong association with underweight.

Children of poor families with a monthly income less than 15,000 PKR were more prone to wasting than those with a relatively higher monthly income (15,000–35,000 PKR). This result is consistent with previous research that highlights a strong relationship between poverty and malnutrition [25]. Monthly family income can impact food intake, malnutrition, and limited resources for low-income families [26]. Financial limitations often make it difficult for people to obtain nutritious food, medical care, and a healthy standard of living, which affects the nutrition of children [27].

Undernutrition has been strongly associated with complementary feeding practices. A higher rate of undernutrition was observed in children who received inadequate complementary feeding. This finding is in alignment with other research emphasizing the need for adequate and timely introduction of complementary foods in promoting optimal growth and development [28]. Inappropriate complementary feeding and weaning practices lead to chronic malnutrition in children.

Improving infant and young child feeding practices is crucial for improving nutrition, health, and development in children aged 0–22 months, ultimately impacting child survival [29]. The WHO’s guidelines recommend complementary feeding for infants and young children aged 6–22 months in low-, middle-, and high-income countries [30]. Breast milk or infant formula alone is insufficient to meet the infant’s energy needs or offer enough amounts of specific nutrients such as protein, zinc, iron, and fat-soluble vitamins from that age [31]. The WHO Global Strategy for Infant and Young Child Feeding recommends introducing solid, semisolid, and soft foods at six months of age, while continuing breastfeeding, to prevent malnutrition [32].

Children who have a balanced diet or enough food have a positive tendency towards healthy development and growth. Malnutrition results from inappropriate weaning procedures and can significantly impair nutritional status [4]. Breastfeeding for the first 24 months of a child’s life, together with complementary feeding, ensures that they receive a sufficient amount of nutrients and a balanced diet, both of which are essential to the child’s growth and the prevention of malnutrition [22]. Mother’s milk alone cannot meet the nutritional requirements of growing children, so breastfeeding should start at birth and continue until the child’s second birthday in combination with complementary feeding to prevent acute and chronic malnutrition [22, 33, 34]. Due to the lack of vital nutrients needed for children’s healthy growth and development, continuing to breastfeed without complementary feeding after six months of age may increase the risk of malnutrition [22].

Malnutrition, both acute and chronic, can result from inappropriate and nonexclusive breastfeeding. According to research conducted in Pakistan, 45.8% of mothers started breastfeeding their children as soon as they were born, and 48.4% of infants were exclusively breastfed [5]. Because the mother’s milk contains an appropriate proportion of the nutrients the infant needs, exclusive nursing should begin within the first hour of delivery [22, 35]. Malnutrition in children caused by infectious diseases could also affect their growth and development [36, 37]. Inflammation increased the likelihood of stunting and malnutrition in children with a history of TB [36].

Compared to children with one or two siblings, children with three or more siblings are more likely to be stunted. Increased family size may affect food availability, resulting in underweight or stunted children [4, 33, 34]. Acute or chronic malnutrition is more likely to develop in children with birth intervals of less than two years [33]. The resources needed to appropriately feed every child become more scarce as the number of children under five in a family increases [4, 37, 38].

The healthy growth and development of newborns and young children are significantly influenced by the education of mothers. The majority of the mothers in the present study were uneducated, and the children they raised had higher nutrient deficiencies. Stunting and wasting may be avoided by an educated mother, as she will be more aware of her child’s nutritional requirements [35].

The cross-sectional design of this study has certain drawbacks. The study’s small sample size and limited coverage in one district limit its potential for strong results, suggesting the need for longitudinal research across multiple districts. Anthropometric measurements were carried out by qualified individuals, which supports the integrity of the data. A deeper understanding of the dynamic nature of a child’s growth and development could be achieved by carefully examining children’s nutritional status.

## Conclusion

The study reveals that age and mid-upper arm circumference are key determinants of weight-for-height and weight-for-age in children under five years, emphasizing the need for interventions targeting specific age groups and practical measures to assess nutritional status. Weaning practices and immunization status are crucial determinants of child development, emphasizing the need for comprehensive health and nutrition programs. Income per month’s importance varies, suggesting that economic context may influence child undernutrition and requires further investigation.

The Boruta analysis revealed that factors such as gender, parasite history, and exclusive breastfeeding are less influential in predicting undernutrition in children. These findings can guide policymakers, healthcare professionals, and researchers in developing targeted strategies to combat undernutrition in children.

## Data Availability

Data are available from the corresponding author upon reasonable request.

## Abbreviations

CI: Confidence interval
CMAM: Community Management of Acute Malnutrition
EPI: Expanded Program of Immunization
PKR: Pakistani rupee
SD: Standard deviation
TB: Tuberculosis
WHO: World Health Organization
